# Maternal Fish Oil Supplementation in Pregnancy: A 12 Year Follow-Up of a Randomised Controlled Trial

**DOI:** 10.3390/nu7032061

**Published:** 2015-03-20

**Authors:** Suzanne Meldrum, Janet A. Dunstan, Jonathan K. Foster, Karen Simmer, Susan L. Prescott

**Affiliations:** 1 Centre for Neonatal Research and Education, School of Paediatrics and Child Health, Faculty of Medicine, Dentistry and Health Sciences, University of Western Australia, Crawley 6009, Australia; E-Mails: dunstan.jan@gmail.com (J.A.D.); jonathan.foster@uwa.edu.au (J.K.F.); karen.simmer@uwa.edu.au (K.S.); susan.prescott@uwa.edu.au (S.L.P.); 2 School of Psychology and Social Science, Faculty of Health, Engineering and Science, Edith Cowan University, Joondalup 6027, Australia; 3 School of Psychology and Speech Pathology, Faculty of Health Sciences, Curtin University, Bentley 6102, Australia; 4 Telethon Kids Institute, University of Western Australia, Perth 6008, Australia

**Keywords:** fish oil, pregnancy, cognition, follow-up

## Abstract

A number of trials have been undertaken to assess whether the intake of omega-3 long-chain polyunsaturated fatty acids (*n*-3 LCPUFA) during pregnancy can influence the neurological development of the offspring, yet no consensus from these trials has been reached. We aimed to investigate the long-term effects (12 years) of fish oil supplementation in pregnancy on neurodevelopment, including cognition, language and fine motor skills. In a follow up of a previously published randomised controlled trial of 98 pregnant women, their children were assessed at 12 years of age using a battery of neurodevelopmental assessments. Fifty participants were assessed at 12 years, with 25 participant’s mothers receiving fish oil supplementation, and 25 receiving control capsules. There were no significant differences for any of the assessment measures completed. Our data indicate that fish oil supplementation during pregnancy does not influence the cognition, language or fine motor skills of children in late primary school (12 years of age).

## 1. Introduction

Omega-3 long-chain polyunsaturated fatty acids (*n*-3 LCPUFA) are thought to be essential for normal neurological development, and are derived from the mother during gestation and breastfeeding. There is concern for potential insufficiencies of *n*-3 LCPUFA during critical periods of fetal neurological development, as maternal diets in Western nations are low in *n*-3 LCPUFA. A number of observational studies and randomised controlled trials (RCTs) have been undertaken to assess whether the intake of *n*-3 LCPUFA during pregnancy can influence the neurological development of the offspring, yet no consensus from these trials has been reached. Our previous randomized controlled trial, in which pregnant women were allocated to receive fish oil capsules (2.2 g DHA, 1.1 g EPA per day) or olive oil from 20 weeks gestation until delivery of their baby, demonstrated that children born of mothers randomised to the fish oil group had statistically significantly higher hand-eye coordination at 2½ years when compared to controls [1]. We now report the neurodevelopmental outcomes of this cohort at 12 years. This represents the longest follow-up of a maternal *n*-3 LCPUFA supplementation study to date. Moreover, at this stage of individual development psychometric test data offer greater predictive value with respect to future adult performance.

## 2. Method

The trial methods were previously published [1]. All study procedures were conducted with written informed consent, as approved by the Princess Margaret Hospital (PMH) Human Research Ethics Committee. At 12 years of age, all study participants who had not previously withdrawn from the study were invited to attend an appointment. Assessment consisted of a battery of tests of cognitive function, language, behaviour and fine-motor control; testing was conducted at the Children’s Clinical Research Facility at Princess Margaret Hospital (CCRF) and performed by a single assessor who was blinded to group allocation (October 2012-December 2013). The primary outcome was Full-Scale IQ measure of the Wechsler Intelligence Scale for Children-IV (WISC-IV) [2]; and secondary outcomes included subtest scores on the WISC-IV, the Child Behaviour Checklist (CBCL, both parent and child forms) [3], the Beery-Buktenica Developmental Test of Visual-Motor Integration (TVMI) [4] and the Children’s Communication Checklist [5]. Information about children’s allergic disease and metabolic health and a blood sample were collected as part of ongoing parallel study. Phospholipid fatty acid composition was measured in erythrocyte cell membranes on available samples according to a previously published method ([6]). Statistical analyses were completed using SPSS Version for PC (Version 20). Analyses were completed on an intention-to-treat basis for participants who consented to follow-up. Independent group’s *t*-test and linear regression was used for data which were normally distributed, while the Mann-Whitney U test was used for data which could not be normalised. Fatty acid data was analysed using multivariable linear regression. Statistical significance was assessed at the 2-sided *p* ≤ 0.05 level. Adjustments were made for parity, sex and maternal education.

## 3. Results

### 3.1. Population Characteristics

**Figure 1:**
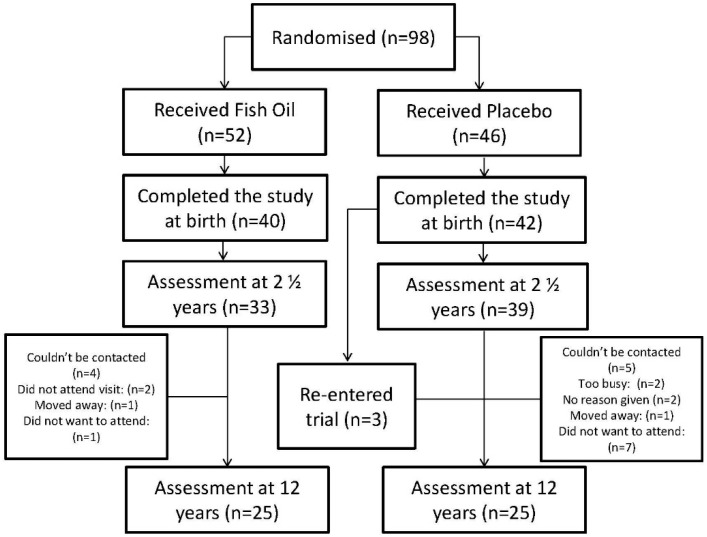
Trial design and participant retention.

Of the 72 participants who completed the previous follow-up at 2½ years of age, 50 participants attended the present follow-up at 12 years of age. An additional three participants who did not attend the 2½ year visit did attend the 12 year assessment. In total, there were therefore 25 participants in each treatment group). This represents a loss of 22 participants from the previous follow-up (see Figure 1). Although loss to follow-up is typical of that reported in many randomised control studies [7], the reduction in power requires cautious interpretation of the findings. The number and proportion of children who had received the fish oil supplement and the placebo were equal, and there were no major differences in the baseline characteristics of the children who were lost to follow up compared with those who returned aged 12 years, with the exception of birth weight (Table S1). A significant difference was observed between the participants who did not attend and the original cohort for birth weight (with the cohort who completed the 12 year visit having a mean lower birth weight than the complete cohort). However, as both groups had means within the normal range for birth weight, this variable was not controlled for in the present statistical analyses.

### 3.2. Neurodevelopmental Finding

At 12 years of age, there were no significant differences between the groups for the primary or secondary analysis, either using adjusted (*i.e*., relative to normative data) or unadjusted data (See Table 1).

**Table 1:** Mean composite scores and percentile ranks for both supplementation groups *.

Measure	Mean Composite Score	Mean Composite Scores	Significance	Adjusted Significance
**WISC-IV Composite**	**Fish Oil (*n* = 25)**	**Placebo (*n* = 25)**		
Verbal Comprehension	105.2 (13.7)	107.1 (12.2)	0.618	0.738
Perceptual Reasoning	110.2 (12.1)	109.6 (9.9)	0.859	0.667
Working Memory	101.9 (11.7)	99.6 (7.8)	0.420	0.522
Processing Speed	107.0 (13.0)	104.4 (11.7)	0.455	0.537
Full Scale IQ	108.6 (12.2)	107.6 (9.9)	0.762	0.670
**Beery-Buktenika TVMI**	**Fish Oil (*n* = 24)**	**Placebo (*n* = 23)**		
Beery VMI Standard Score	104.4 (9.0)	103.2 (9.9)	0.655	0.232
Beery VMI Percentiles	60.9 (20.8)	57.6 (20.1)	0.583	0.192
**CBCL Parent-Report**	**Fish Oil (*n* = 23)**	**Placebo (*n* = 25)**		
Internalising Behaviours	46.7 (8.7)	48.3 (9.5)	0.541	0.760
Externalising Behaviours	46.4 (7.7)	42.6 (7.2)	0.090	0.051
Total Behaviours Score	46.4 (8.2)	47.2 (7.4)	0.796	0.890
Total Competence Score	52.2 (8.6)	52.4 (12.9)	0.634	0.540
**CBCL Child-Self Report**	**Fish Oil (*n* = 22)**	**Placebo (*n* = 25)**		
Internalising Behaviours	47.4 (9.6)	47.0 (6.5)	0.940	0.409
Externalising Behaviours	42.9 (9.6)	42.5 (6.9)	0.974	0.550
Total Behaviours Score	47.5 (9.2)	45.1 (7.5)	0.527	0.141
Total Competence Score	53.5 (9.6)	50.5 (11.9)	0.386	0.358
**Children’s Communication Checklist (CCC-2)**	**Fish Oil (*n* = 22)**	**Placebo (*n* = 25)**		
GCC Percentile Rank	54.5 (27.6)	52.3 (27.5)	0.773	0.866

* Scores are represented as mean (SD) unless otherwise stated.

### 3.3. Fatty Acid Analysis

At 12 years of age, a total of 43 participants (24 from the fish oil group, 19 from placebo) provided blood samples for fatty acid analyses. The *n*-3 LCPUFA measurements taken at 12 years were not significantly correlated with those from cord blood following supplementation (Table 2). Further, the fish oil and placebo groups did not significantly differ according to their *n*-3 LCPUFA status at 12 years (Table 3). This likely demonstrates the influence of *n*-3 LCPUFA consumption during the intervening 12 years following supplementation.

Interestingly, when erythrocyte DHA measurements at 12 years of age were associated with neurodevelopmental measures at 12 years (*n* = 40; 23 from fish oil group, 17 from placebo), significant positive associations were observed for several composite scores of the WISC-IV, including the Full Scale IQ. This effect persisted following adjustment for sex, maternal education and length of breastfeeding during infancy (Table 4).

**Table 2:** Correlation between *n*-3 LCPUFA in cord blood (post supplementation) and at 12 years (*n* = 35).

Fatty Acid	Cord Blood	12 Years	Pearson Correlation
EPA (20:5*n*-3) (*n* = 35)	0.851 (0.63)	0.809 (0.22)	−0.179, *p* = 0.304
DHA (22:6*n*-3) (*n* = 43)	8.84 (1.7)	4.51 (0.88)	0.290, *p* = 0.059

**Table 3:** Difference in *n*-3 LCPUFA at 12 years according to supplementation group during pregnancy.

Fatty Acid	Fish Oil (*n* = 24)	Placebo (*n* = 19)	Significance
EPA (20:5*n*-3)	0.754 (0.16)	0.880 (0.27)	0.082
DHA (22:6*n*-3)	4.65 (0.72)	4.34 (1.0)	0.266

**Table 4:** Association between DHA and neurodevelopmental outcome at 12 years ^.

Measure	Unadjusted Analysis		Adjusted Analysis	
	β	Significance	β	Significance
**WISC-IV Composite**				
Verbal Comprehension	0.293	0.067	0.329	0.057
Perceptual Reasoning	0.466	0.002 **	0.332	0.042 *
Working Memory	0.372	0.018 *	0.411	0.016 *
Processing Speed	0.441	0.004 **	0.332	0.042 *
Full Scale IQ	0.522	0.001 **	0.468	0.004 **
**Beery-Buktenika TVMI**				
Beery VMI Standard Score	0.046	0.784	−0.109	0.571
Beery VMI Percentiles	0.037	0.823	−0.121	0.497
**CBCL Parent-Report**				
Internalising Behaviours	−0.026	0.878	−0.005	0.980
Externalising Behaviours	−0.003	0.985	0.031	0.869
Total Behaviours Score	−0.143	0.391	−0.030	0.866
Total Competence Score	0.076	0.649	−0.051	0.780
**CBCL Child-Self Report**				
Internalising Behaviours	−0.255	0.128	−0.149	0.430
Externalising Behaviours	−0.181	0.283	−0.115	0.520
Total Behaviours Score	−0.232	0.168	−0.093	0.611
Total Competence Score	0.134	0.428	0.173	0.380
**Children’s Communication Checklist (CCC-2)**				
GCC Percentile Rank	0.006	0.972	−0.076	0.696

^ Adjusted statistics controlled for maternal education, duration of breastfeeding in the first twelve months and child gender; * *p* < 0.05; ** *p* < 0.01.

## 4. Discussion

Our data indicate that *n*-3 LCPUFA supplementation during pregnancy does not influence the cognition, language or fine motor skills of children in late primary school (12 years of age). The significant differences observed in hand-eye coordination at 2½ years of age could no longer be detected, and may have been diluted by other environmental factors. Alternatively, the findings reported at aged 2 ½ years may have represented a type I statistical error. This is in line with other recent findings published this year, including a large meta-analysis completed by Gould *et al*. [8], and the results of a large long-term follow up study completed by Makrides and colleagues [9].

Fatty acid analyses indicate that the supplementation effect of raising *n*-3 LCPUFA status in cord blood was no longer present at 12 years. This suggests that the nutritional intake of the participants in the intervening years diluted the effect of the supplementation during pregnancy. Yet, akin to other reported correlational studies, current *n*-3 LCPUFA intake was important for neurological performance [10,11]. These results need to be interpreted with caution however, considering the low sample size relative to other published studies in this field.

This current study has strength in being the longest follow-up of a maternal *n*-3 LCPUFA supplementation trial to date. Furthermore, this study used a well validated assessment protocol. However, there was a high attrition rate in our study, which was largely due to our inability to contact participants or due to a lack of interest in the study participants to attend. The reduced number of participants in this follow-up limits the statistical power of the study to identify treatment-related differences. Indeed, a near significant finding for externalising behaviours as reported by their parents may have reached significance in a larger cohort.

## 5. Conclusions

In conclusion, our data suggests that *n*-3 LCPUFA supplementation in pregnancy is ineffective for enhancing later childhood development in healthy children born at term. However, further studies with larger sample sizes are required to obtain more conclusive results.

## Supplementary Files

Supplementary File 1
