# Supplementary material for: Maternal Fish Oil Supplementation in Pregnancy: A 12 Year Follow-Up of a Randomised Controlled Trial
Source: Nutrients. 2015 Mar 20;7(3):2061–7. doi: 10.3390/nu7032061 (PMC4377900; doi:10.3390/nu7032061)
Supplement: Supplementary File 1 [file nutrients-07-02061-s001.docx]

**Supplementary Information**

**Table S1.** Population characteristics of dropouts at 2.5 and 12 years compared with children completing the intervention and follow-up.

| **Characteristic** | **Completed Intervention  (*n* = 83)** | | **Year 2.5 Dropouts  (*n* = 11)** | | **Year 12 Dropouts  (*n* = 33)** | |
| --- | --- | --- | --- | --- | --- | --- |
|  | ***N* (%)** | | ***N* (%)** | ***P* value** | ***N* (%)** | ***P* value** |
| **At birth** |  | |  |  |  |  |
| Fish Oil Group | 40/82 (48%) | | 7/11 (64%) | 0.219 | 15/33 (45%) | 0.428 |
| Boys | 39/83 (47%) | 6/11 (54%) | | 0.414 | 17/33 (51%) | 0.327 |
| Maternal education  (% > year 12) | 58/83 (70%) | 6/11 (54%) | | 0.065 | 20/33 (61%) | 0.106 |
| Gestation, Days | 274.4 (7.9) | 269.7 (7.0) | | 0.047* | 272.5 (8.4) | 0.087 |
| Weight (g) | 3465.4 (355) | 3447.3 (292.9) | | 0.559 | 3352.3 (399.1) | 0.035* |
| Length (cm) | 50.1 (1.9) | 50.0 (2.1) | | 0.973 | 50.5 (1.8) | 0.064 |
| Head circumference (cm) | 34.9 (1.3) | 34.7 (1.2) | | 0.550 | 34.9 (1.3) | 0.912 |
| Parity (% first born child) | 39/83 (47%) | 3/11 (27%) | | 0.139 | 13/33 (39%) | 0.184 |
| Maternal age (year) | 31.8 (3.7) | 31.3 (4.2) | | 0.505 | 31.5 (3.8) | 0.695 |
| Apgar | 8.3 (.82) | 8.3 (.86) | | 0.924 | 8.5 (0.66) | 0.265 |
| Cord Blood Eicosapentanoic Acid | 0.826 (.63) | 1.0 (.65) | | 0.216 | 0.885 (0.66) | 0.118 |
| Cord Blood Docosahexaenoic Acid | 8.69 (1.8) | 8.8 (1.9) | | 0.683 | 0.733 (0.58) | 0.316 |
| **Early Childhood** |  |  | |  |  |  |
| Breast fed ever (% yes) | 77/82 (94%) | 11/11 (100%) | | 0.477 | 31/32 (97%) | 0.348 |
| Breastfeeding duration in 1st year (days) | 223.1 (139.8) | 207.8 (134.5) | | 0.564 | 212.0 (131.0) | 0.443 |
| Atopy at 1 year (% affected) | 38/74 (51%) | 6/7 (86%) | | 0.062 | 17/28 (61%) | 0.154 |
| Height at 2.5 years | 93.5 (4.2) | 90.4 (4.9) | | 0.191 | 92.8 (4.5) | 0.370 |
| Weight at 2.5 years | 14.3 (1.9) | 15.0 (4.0) | | 0.921 | 14.4 (2.7) | 0.604 |
| Head circumference at 2.5 years | 49.6 (1.7) | 49.8 (2.4) | | 0.771 | 49.5 (1.8) | 0.552 |

* *p* < 0.05; ** *p* < 0.01.

© 2015 by the authors; licensee MDPI, Basel, Switzerland. This article is an open access article distributed under the terms and conditions of the Creative Commons Attribution license (http://creativecommons.org/licenses/by/4.0/).
